# Evaluation of Halogenated Coumarins for Antimosquito Properties

**DOI:** 10.1155/2014/189824

**Published:** 2014-12-25

**Authors:** Venugopala K. Narayanaswamy, Raquel M. Gleiser, Kabange Kasumbwe, Bandar E. Aldhubiab, Mahesh V. Attimarad, Bharti Odhav

**Affiliations:** ^1^Department of Pharmaceutical Sciences, College of Clinical Pharmacy, King Faisal University, Al-Ahsa 31982, Saudi Arabia; ^2^Department of Biotechnology and Food Technology, Durban University of Technology, Durban 4001, South Africa; ^3^CREAN-IMBIV (CONICET-UNC), Facultad de Ciencias Agropecuarias, and FCEFyN, Universidad Nacional de Córdoba, Avenida Valparaíso s/n, 5016 Córdoba, Argentina

## Abstract

Mosquitoes are the major vectors of parasites and pathogens affecting humans and domestic animals. The widespread development of insecticide resistance and negative environmental effects of most synthetic compounds support an interest in finding and developing alternative products against mosquitoes. Natural coumarins and synthetic coumarin analogues are known for their several pharmacological properties, including being insecticidal. In the present study halogenated coumarins (3-mono/dibromo acetyl, 6-halogenated coumarin analogues) were screened for larvicidal, adulticidal, and repellent properties against *Anopheles arabiensis*, a zoophilic mosquito that is one of the dominant vectors of malaria in Africa. Five compounds exerted 100% larval mortality within 24 h of exposure. All coumarins and halogenated coumarins reversibly knocked down adult mosquitoes but did not kill them after 24 h of exposure. Repellent properties could not be evidenced. Five compounds were considered potential larvicidal agents for further research and development, while adulticidal activity was considered only mild to moderate.

## 1. Introduction

Mosquitoes are the major vectors of parasites and pathogens that cause malaria, filariasis, dengue fever, yellow fever, Japanese encephalitis, and other fevers affecting humans and domestic animals [1]. Vector control programs mainly use four classes of chemical insecticides: organochlorines, organophosphates, carbamates, and pyrethroids. Bacterial insecticides and insect growth regulators have also become more widely used in recent years. However, use of chemicals on a vast and increasing scale has led to the widespread development of resistance as a result of selection for certain genes [2], and some species have even become resistant to multiple insecticides [3]. Mosquito resistance to at least one insecticide used for malaria control has been identified in 64 countries [3]. Besides, synthetic organic insecticides, such as those currently used to control mosquitoes, affect nontarget organisms and result in negative environmental effects [4]. Thus, there is an interest in the finding and development of alternative antimosquito products.

Coumarins (2*H*-1-benzopyran-2-one) are a class of phenolic substances found as secondary metabolites from plants, bacteria, and fungi, widely used as additives in food, perfumes, cosmetics, pharmaceuticals, optical brighteners, and dispersed fluorescent. Natural coumarins are known for their several pharmacological properties and have been recently reviewed by Venugopala et al. [5], also reported for synthetic coumarin analogues, such as analgesic, anti-inflammatory [6, 7], anticoagulant [8], antibacterial [9–11], antifungal [12], antiviral, anticancer [13], antihypertensive [14], antitubercular [15], antihyperglycemic [16], and antioxidant [17] properties. Warfarin is a synthetic coumarin analogue (known as Coumadin) that is used as an anticoagulant and is commercially available in the market with a trade name Coumadin. Essential oils and solvent extracts of plants containing coumarin have shown promising properties against mosquitoes. For example, coumarin extracted from southernwood (*Artemisia abrotanum* L.) and essential oil of carnation flowers (*Dianthus caryophyllus* L.) exerted a repellent effect against yellow fever mosquitoes (*Aedes aegypti* L.) and ticks (nymphs of* Ixodes ricinus* L.) [18]. Eight coumarin derivatives obtained from hexane extractions of the roots of* Esenbeckia grandiflora* Mart. were effective larvicides against* Ae. aegypti* [19]. The coumarin derivative pachyrrhizine, a compound from tubers of* Neorautanenia mitis*, showed larvicidal and adulticidal activities against* Anopheles gambiae* and* Culex quinquefasciatus* Say that were comparable to deltamethrin and alpha-cypermethrin, two standard mosquitocides [20]. A component of* Tagetes lucida* Cav. hexane extract, 7-methoxy coumarin, showed larvicidal activity against* Ae*.* aegypti* [21]. The linear furanocoumarin imperatorin and the coumarin osthole extracted from* Cnidium monnieri* (L.) Cusson fruit were effective against larvae of* Culex pipiens pallens* Coquillett and* Ae. aegypti* and against* C. p. pallens* larvae resistant to various insecticides, suggesting that these coumarins and the pyrethroid and organophosphate insecticides do not share a common mode of action or elicit cross-resistance [22]. In this context, and in continuation of our search for novel chemical agents with antimosquito properties [23, 24], in the present study we undertake the screening of halogenated coumarins (3-mono/dibromo acetyl, 6-halogenated coumarin analogues) for larvicidal and repellent properties against* Anopheles arabiensis*, one of the dominant vectors of malaria in Africa [25].

## 2. Materials and Methods

### 2.1. Chemicals Tested

**Figure 1:**
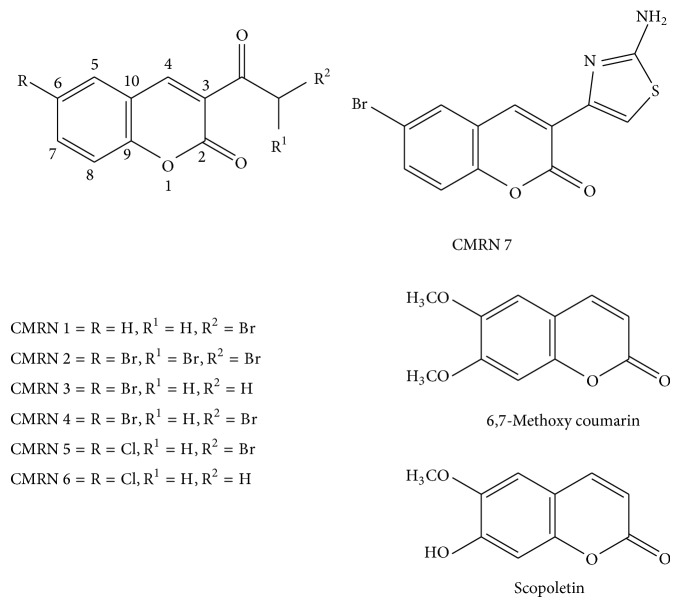
Synthetic halogenated coumarin compounds CMRN 1–CMRN 7, 6,7-dimethoxy coumarin, and scopoletin tested for antimosquito properties.

The test compounds halogenated coumarin analogues (CMRN 1–CMRN 7) were synthesized as described previously [6, 7, 9–11, 26–28], using chemicals from Aldrich and Merck chemical company without further purification. Compounds 6,7-methoxy coumarin and scopoletin were obtained from Sigma-Aldrich. The products assessed were 3-(2-bromoacetyl)-2*H*-chromen-2-one (CMRN 1) [7, 11] and 6-bromo-3-(2,2-dibromoacetyl)-2*H*-chromen-2-one (CMRN 2) [28]; 3-acetyl-6-bromo-2*H*-chromen-2-one (CMRN 3) [6], 6-bromo-3-(2-bromoacetyl)-2*H*-chromen-2-one (CMRN 4) [9], and 3-(2-bromoacetyl)-6-chloro-2*H*-chromen-2-one (CMRN 5) [10]; 3-acetyl-6-chloro-2*H*-chromen-2-one (CMRN 6) [26]; 3-(2-aminothiazol-4-yl)-6-bromo-2*H*-chromen-2-one (CMRN 7) [27]; 6,7-methoxy coumarin; and scopoletin (Figure 1). The physicochemical characteristics of the halogenated coumarin analogues (CMRN 1–CMRN 7) are summarized in Table 1.

### 2.2. Larvicidal Activity

The* Anopheles arabiensis* used were from a colonized strain from Zimbabwe which had been reared according to the WHO (1975) guidelines [1] in an insectary simulating the temperature (27.5°C), humidity (70%), and lighting (12/12) of a malaria endemic environment. One mL of test compound (1 mg/mL) was added to distilled water (250 mL) producing a final concentration of 4 *µ*g/mL. Thirty 3rd instar larvae were placed in the container. A negative control was set up using a solvent (acetone) and distilled water and a positive control included Temephos (Mostop; Agrivo), an effective emulsifiable organophosphate larvicidal used by the malarial control program. Each container was monitored for larval mortality at 24 h intervals for a period of three days and fed specially made cat food with reduced oil/fat content at regular intervals. Bioassays were triplicated. The percentage mortality was calculated relative to the initial number of exposed larvae. The larvicidal results are tabulated in Table 2.

### 2.3. Insecticidal Activity

Insecticidal activity assessment was conducted by exposing susceptible adult mosquitoes to a treated surface, in accordance with WHO protocol (1975) [1]. One mL of test compound solution (1 mg/mL) was sprayed onto a clean, dry, nonporous ceramic tile using a precalibrated Potter's Tower apparatus [8]. The tiles were then air-dried and the assay was initiated within 24 h of spraying, by fixing a cone over the sprayed tile and introducing thirty non-blood-fed, 2–5-day-old susceptible adult* A. arabiensis* mosquitoes into the cone. The effect of the test compounds was measured by determining the knockdown rate, which was based on temporary paralysis of the mosquitoes during a 60 min exposure period, and mortality 24 h postexposure. Deltamethrin (15 g/L; K-Othrine) was used as a positive control and acetone as a negative control. All bioassays were triplicate to ensure validity of results.

### 2.4. Repellence Assays

Repellent activity was assessed by topical application of the compound to skin and subsequent exposure of the treated areas of skin to unfed female mosquitoes. Ethical approval for the use of* Mastomys coucha* in these trials was approved from the Medical Research Council's Ethics Committee for Research on Animals. Adult* Mastomys* rodents were weighed individually and injected intraperitoneally with the correct concentration of sodium pentobarbital in comparison to the weight of the animal. The anesthetized rodents were then shaved on the ventral surface and a test compound (1 mL) was applied to each rodent's abdomen. Acetone was used as a solvent for the preparation of stock solution (1 mg/mL). Laboratory grade DEET (IUPAC:* N*,*N*-Diethyl-3-methylbenzamide) was used as the positive control and plain acetone was used as negative control.

Paper cups (500 mL) were modified by replacing the base of the cup with mosquito netting held in place with a rubber band and covering the mouth of the cup with transparent plastic film. Thirty unfed 4-day-old* A. arabiensis* females were introduced into the cup that was held in contact with the treated ventral surface of each rodent. Mosquito activity was observed through the transparent plastic film. After a period of 2 min, the numbers of mosquitoes probing were recorded. The cups holding the mosquitoes were removed and mosquitoes were then observed for 24 h. All tests were triplicated. The rodent was then returned to the animal facility and allowed to recover from anaesthetic. No adverse reactions to the applied components were observed on any of the* Mastomys* rodents during the 3 days they were monitored.

Repellence of the extracts was calculated using the following formula;

(1) Percentage  mosquitoes  repelled =Number  repelledNumber  introduced×100.

### 2.5. Statistical Analysis

Generalized linear models assuming a Gaussian distribution were used to determine differences between treatments registered in larval mortality (larvicidal assays), adulticidal effects, and knockdown (in repellence assays). LSD Fisher test was used for post hoc analyses. In all cases, a value of *P* < 0.05 was considered statistically significant.

## 3. Results and Discussion

The title compounds obtained were in good yields (87–98%) and characterization was completed by GCMS analysis. Purity of the compounds was confirmed by HPLC and it was more than 99%. *c* Log⁡ *P* of the compounds was calculated using ChemBioDraw Ultra 13.0 v and values were in the range of 1.8693–3.4753.

There was a significant effect of treatment on larval mortality (*P* < 0.001) (Table 2). The highest activity was detected with CMRN 1, CMRN 2, CMRN 4, CMRN 5, and CMRN 7 showing close to 100% mortality after 24 h of exposure, which was the same as for the positive control Temephos. Compounds CMRN 3, CMRN 6, scopoletin, and 6,7-methoxy coumarin showed a statistically lower mortality that was equivalent to the negative control.

The promising larvicidal activity of CMRN 1, CMRN 2, CMRN 4, CMRN 5, and CMRN 7, which was comparable to the positive control Temephos, may be attributed to the presence of electron withdrawing halogen atoms (bromine and chlorine) on acetyl group at the third and sixth positions of the coumarin nucleus. Larvicidal and ovicidal activity of 4-methyl-7-hydroxy coumarin derivate against vectors* Aedes aegypti* and* Culex quinquefasciatus* have also been attributed to bromine atoms present at C-5 and C-8 positions [29]. Coumarin and mainly furanocoumarins can alter the detoxication capability of an organism, by reversibly or irreversibly inhibiting cytochrome P450 detoxication enzymes [30, 31].

On adulticidal assays, adult mosquito mortality of positive control K-Othrine showed 100% knockdown/mortality from the first 60 min of exposure, while the natural coumarin, synthetic compounds, and negative control did not knockdown mosquitoes throughout the 24 h observation period.

All components tested for repellence (except the controls) knocked down mosquitoes within the 2 min exposure time, and CMRN 1, CMRN 2, CMRN 5, CMRN 6, 6,7-methoxy coumarin, and scopoletin were the most potent, knocking approximately 100% of them (*P* < 0.001) (Table 2). However, 24 h after exposure, all mosquitoes recovered. The few mosquitoes exposed to the coumarin analogues that were not knocked down did not attempt to bite; because of the low number of mosquitoes remaining active, no further statistical analyses were carried out.

An insect immobilization effect of coumarins has been reported for other insects [32, 33], and a slowly developing paralysis eventually leading to death has been a major feature of insect poisoning by coumarins such as surangin B [32]. Bioenergetic disruption of muscle has been determined as a prominent mechanism underlying the insecticidal action of surangin B [34]. Moreover, homology modeling and docking studies indicate that coumarin, as well as other terpene compounds, may act as acetylcholinesterase inhibitors and can block the octopamine receptor pathway and thus be neurotoxic against mosquitoes [35]. However, knockdown of mosquitoes resulting from short exposure to 3-mono/dibromoacetyl-6-halogenated coumarin analogues CMRN 1–CMRN 7 was fast (2 min or less) and reversible, and no adulticidal effects were detected from 60 min exposure. This may be partly explained by differences in experimental design, since topical application and injection were common procedures in previous reports on insecticidal properties [32, 33]. Besides mortality, coumarins may exert other negative effects on insect populations, such as decreasing their reproductive potential [36], which were not assessed in the present study.

Because no (active) mosquitoes were observed attempting to bite the rodent treated with coumarin analogues, further assessment of the repellent properties of these compounds after different treatment times and concentrations merits further testing.

## 4. Conclusions

The present study evaluates 3-mono/dibromoacetyl-6-halogenated coumarin analogues CMRN 1–CMRN 7, scopoletin, and 6,7-methoxy coumarin for larvicidal and repellent effects against the malaria vector* A. arabiensis*. Compounds CMRN 1, CMRN 2, CMRN 4, CMRN 5, and CMRN 7 were considered potential larvicidal agents for further research and development, because these compounds exerted close to 100% mortality within 24 h of exposure. Adulticidal activity on the other hand was considered negligible and repellence should be further explored. Of particular interest are the bromo- and chloroanalogues of CMRN 2, CMRN 4, and CMRN 5 that have potential to be used to prevent and control malaria by controlling the vector* A. arabiensis.*

## Figures and Tables

**Table 1:** Physicochemical characteristics of 3-mono/dibromoacetyl-6-halogenated coumarin analogues CMRN 1–CMRN 7.

Code	M. F (M. Wt.)	Yield (%)^a,b^	m.p. (°C) reported	m.p. (°C) found	Mass (*m*/*z*)	*c*log⁡*P* ^c^
CMRN 1	C_11_H_7_BrO_3_ (265)	96	120–122	121	266 (*M* + 1)	1.4023
CMRN 2	C_11_H_5_Br_3_O_3_ (421)	95	146–148	147	422 (*M* + 1)	3.4753
CMRN 3	C_11_H_7_BrO_3_ (265)	98	220–222	221	266 (*M* + 1)	2.0193
CMRN 4	C_11_H_6_Br_2_O_3_ (343)	95	204–206	205	344 (*M* + 1)	2.2723
CMRN 5	C_11_H_6_BrClO_3_ (299)	94	180–182	181	300 (*M* + 1)	2.1223
CMRN 6	C_11_H_7_ClO_3_ (222)	95	218–220	219	223 (*M* + 1)	1.8693
CMRN 7	C_12_H_7_BrN_2_O_2_S (321)	87	210–212	211	322 (*M* + 1)	2.6992

^a^All of the products were characterized by spectral and physical data.

^b^Yields were on isolated basis.

^c^ *c*log⁡*P* was calculated using ChemBioDraw Ultra 13.0 v.

**Table 2:** Mortality of *Anopheles arabiensis* larvae exposed to coumarins and halogenated coumarins at 4 *μ*g/mL and their negative (acetone) and positive (Temephos) controls and adult knockdown activity after 2-minute exposure to halogenated coumarins at 1000 *μ*g/mL and their negative (acetone) and positive (DEET) controls against repellence assays (adjusted means and standard errors).

Compound code	Larval mortality	Knocked down
CMRN 1	98.9 ± 3.1^a^	100.0 ± 10.9^a^
CMRN 2	97.8 ± 3.1^a^	96.7 ± 10.9^ab^
CMRN 3	1.1 ± 3.1^b^	91.1 ± 10.9^b^
CMRN 4	98.9 ± 3.1^a^	91.1 ± 10.9^b^
CMRN 5	97.8 ± 3.1^a^	97.8 ± 10.9^ab^
CMRN 6	1.1 ± 3.1^b^	95.6 ± 10.9^ab^
CMRN 7	98.9 ± 3.1^a^	81.1 ± 10.9^c^
6,7-Methoxy coumarin	3.3 ± 3.1^b^	96.7 ± 10.9^ab^
Scopoletin	0.0 ± 3.1^b^	96.7 ± 10.9^ab^
Acetone (control)	0.0 ± 3.1^b^	0.0 ± 10.9^d^
Temephos	100.0 ± 3.1^a^	
DEET		0.0 ± 10.9^d^

^a–d^Within each column, compounds not sharing a letter differ significantly (*P* < 0.05).
